# Supplementary material for: High-risk fertility behaviours among women of reproductive ages in the Democratic Republic of the Congo: Prevalence, correlates, and spatial distribution
Source: PLoS One. 2023 Mar 16;18(3):e0283236. doi: 10.1371/journal.pone.0283236 (PMC10019681; doi:10.1371/journal.pone.0283236)
Supplement: Attachment — Submitted filename: Review - High-risk fetility behaviors....pdf [file pone.0283236.s001.pdf]

"***High-risk fertility behaviors among women of reproductive ages in the Democratic Republic of the Congo: Prevalebce, Correlates and Spatial distribution***"

By Zacharie Tsala Dimbuene, Zemenu Tadesse Tessema, and Soziac Elise Wang Sonne

Reviewed by Bluette Arcady Mongoue, October 2022

This is an important empirical paper that should benefit from wide diffusion; more particularly in the context of Africa south of the Sahara where the number of deaths following high-risk pregnancies or neonatal / perinatal deaths remain greatly high compared to the rest of the world. The study is relevant in such context where health care is still quite precarious; mainly in regions that have witnessed political instability and violence.

As the authors mentioned, the paper is not the first to investigate the correlates and implications of High fertility risk behaviors among women of reproductive age. However, it differs from the existing literature in that it identifies the geospatial distribution of risky fertility decision in the Democratic Republic of the Congo. This work could therefore constitute a good starting point for the Congolese authorities who would like to limit risky fertility behaviors among Congolese women.

The paper identifies factors that are correlated to women risky fertility behaviors in RDC.

The authors conclude that women's current age significantly increased the odds of high-risk fertility behaviors. As mentioned in the pater, they obtained this result by performing a standard logistic regression of an indicator of high-risk fertility behaviors on age education and other individuals and household characteristics. That indicator of high-risk fertility behavior is clearly correlated to the age of women in their sample (as the indicator take the value one when the current age is greater than 34). However, without contesting the veracity of this result, there is clearly a bias in the impact of the women's age on the probability of presenting a positive risk. What would be the incidence of the mother's age on her probability risk among mothers aged 33 or less?

The manuscript is technically sound, however, as to the geographical distribution of risky behavior, it is difficult to know whether the authors are talking about the Northern region or the Eastern region.

The authors argue that:" the country has been devastated by successive wars and armed conflict since 1996 in its Eastern part. As a consequence, the health system has worsened, especially in the Eastern region of the country. This has certainly increased the prevalence of high-risk fertility behaviors in the country". But their findings reveal that higher risk are mostly prevalent in the northern part of the country. Why are the high spots of fertility risk not in the Eastern region? Which factors do explain the high spots in the Northern part of the country

The literature that studies the relationship between political instability, wars and fertility decisions seems to disagree on the nature of the relationship between armed conflict and fertility choices. On one hand, we have a group of studies that believes that political instability and related violence, not to mention economic uncertainties, negatively affect social returns such as population growth, and so it will logically follow a decline in fertility decisions and therefore less predominant risky fertility behaviors ((Berrebi and Ostwald (2015) , Caldwell (2004); Agadjanian et al (2011), McGinn (2000)). On the other

hand, we have authors who rather think that armed conflicts would be likely to reinforce fertility decisions (Urdal and Che (2013), McGinn et al (2011)…), and thus increase risky fertility behaviors .
This article is not precise enough at this level, since it is difficult to identify if it is in the first or the second group. Indeed, the text is a bit ambiguous. It seems to indicate a strong prevalence of risky fertility behaviors in the North of the country.
This article would therefore stand out from the existing literature by identifying what is happening in the case of the Republic Democratic of Congo in the Eastern region of the country which has been affected by the war. And even more by identifying the mechanisms Through which the violence experienced by this region would reinforce the risky fertility behaviors. Such mechanisms could indeed be interesting targets for policy makers.

References:

Caldwell JC (2004). Social Upheaval and Fertility Decline. *Journal of Family History*, 29(4), 384–406

Agadjanian V, Yabiku T, & Boaventura C (2011). Men's Migration and Women's Fertility in Rural Mozambique. *Demography*, 48(3), 1029–48.

McGinn T (2000). Reproductive Health of War-Affected Populations: What Do We Know? *International Family Planning Perspectives*, 26(4), 174–18

Berrebi C & Ostwald J (2015). Terrorism and Fertility: Evidence for a Causal influence of Terrorism on Fertility. *Oxford Economic Papers*, 67(1), 63–82

McGinn T, Austin J, Anfinson K, Amsalu R, Casey SE, Fadulalmula S, & Yetter M (2011). Family Planning in Conflict: Results of Cross-Sectional Baseline Surveys in Three African Countries. *Conflict and Health*, 5(1), 11. H

Urdal H & Che CP (2013). War and Gender inequalities in Health: The Impact of Armed Conflict on Fertility and Maternal Mortality. *International Interactions*, 39(4), 489–510
