# Supplementary material for: High-risk fertility behaviours among women of reproductive ages in the Democratic Republic of the Congo: Prevalence, correlates, and spatial distribution
Source: PLoS One. 2023 Mar 16;18(3):e0283236. doi: 10.1371/journal.pone.0283236 (PMC10019681; doi:10.1371/journal.pone.0283236)
Supplement: Attachment — Submitted filename: Point-by-point Responses Dec 2022.docx [file pone.0283236.s002.docx]

**PLOS ONE Point-by-point responses**

**Manuscript: PONE-D-22-12958**

Title: “**High-risk fertility behaviors among women of reproductive ages in the Democratic Republic of the Congo: prevalence, correlates and spatial distribution**”

**Editor’s or Journal’s comments or requirements**

1. Please ensure that your manuscript meets PLOS ONE's style requirements, including those for file naming

**Response: The manuscript has been revised accordingly to meet PLOS ONE’s style requirements, and file naming**

2. Thank you for stating the following in your Competing Interests section:

**Response: As suggested, “The authors have declared that no competing interests exist.” has been stated in the “Competing Interests” section**

3. We note that [Figures 4-8] in your submission contain [map/satellite] images which may be copyrighted

**Response: Just to put a bit things in context. Figures 4—8 were produced using authors’ own analyses and shouldn’t be “copyrighted”. Indeed, the paper used Demographic and Health Survey (DHS) conducted in the Democratic Republic of the Congo between November 2013 and February 2014 regarding “data collection”. Along with data, DHS also collected georeferenced information system (GIS) about households and clusters. When researchers plan to do “spatial analyses” using DHS data, they request access to GIS data and can therefore perform the own spatial analyses. In this case, there is no “copyright” issues with any organization. There are many instances of papers using GIS along with DHS data published in PLOS ONE and as far as we know, “copyright” of mapping produced with DHS and GIS data has never been an issue. Hope this clarifies this query.**

**Reviewers’ comments**

**Reviewer #1**

4. Thanks to the authors for studying an important issue. We know sub-Saharan African countries are more vulnerable for public health outcomes for different reasons. I am happy reading the paper it is really good enough and it has value for further investigation in this topic.

**Response: Thanks for this positive comment. The authors appreciate your invaluable time to read and comment on the paper.**

5. However, I have some addressable comments. What is the power of the analysis^[1]^?

**Response: First, it is important to mention that Demographic and Health Surveys (DHS) are nationally-representative household surveys which estimate a wide range of monitoring and impact evaluation indicators in the areas of population, health, and nutrition. Second, these surveys use similar data collection methods within and across countries. As such, the sample sizes within each cluster and stratum were calculated to provide with reliable estimates at sub-national and national levels. In other words, power of analysis was taken into account during the “design” of the surveys and its implementation.**

**To answer specifically the reviewer’s query, we all know that “statistical power” is influenced by four factors, including (*i*) the level of statistical significance (*α*); (*ii*) effect size—the magnitude of the difference between the two sample groups being examined on a specific outcome variable; (*iii*) the variance of the responses to the outcome variable; and (*iv*) the size of the sample. Given these components, this paper is based on a large sample size (*N* = 18,827) of women of reproductive ages. The statistical significance was fixed at the conservative level (*α* = 0.05). in the reporting and to account for the effect size, the paper reported the 95% confidence intervals since the “effect size” is usually unknown and needs to be estimated. With all these elements, the authors believe that all estimates reported in the paper comply with an acceptable power since (*i*) the sample is probabilistic and representative of the entire population, and (*ii*) was calculated to detect differences between groups.**

6. Why did you do the study that is missing in the rationale section? I think you did multilevel modelling but it is showing multivariate please clarify it.

**Response: Nothing in the rationale section and a standard logistic regression was performed. Initially, the authors thought to perform “multilevel logistic regression”. As we know, the starting point for a “justifiable” multilevel modeling is to ensure that there is significant variable at the higher-order of the variables of interest. In this case, a “justified” multilevel modeling relies on a significant variation of high-risk fertility behavior at household/community level. This was not the case (see below). Indeed, the intraclass correlation (ICC) equals 1.2% which means that only 1.2% of chances of having high-risk fertility behavior among married women of reproductive ages in the DRC depends on the between-cluster differences. This also means that multilevel logistic regression is not justified.**

```
. melogit HRFB if select == 1  || v001 :

Fitting fixed-effects model:

Iteration 0:   log likelihood =  -7121.109
Iteration 1:   log likelihood = -7102.4813
Iteration 2:   log likelihood = -7102.4681
Iteration 3:   log likelihood = -7102.4681

Refining starting values:

Grid node 0:   log likelihood =  -7281.997

Fitting full model:

Iteration 0:   log likelihood =  -7281.997  (not concave)
Iteration 1:   log likelihood = -7100.5249
Iteration 2:   log likelihood = -7098.7335
Iteration 3:   log likelihood = -7098.3327
Iteration 4:   log likelihood = -7098.3327

Mixed-effects logistic regression               Number of obs     =     11,497
Group variable:            v001                 Number of groups  =        536

                                                Obs per group:
                                                              min =          7
                                                              avg =       21.4
                                                              max =         37

Integration method: mvaghermite                 Integration pts.  =          7

                                                Wald chi2(0)      =          .
Log likelihood = -7098.3327                     Prob > chi2       =          .
------------------------------------------------------------------------------
        HRFB |      Coef.   Std. Err.      z    P>|z|     [95% Conf. Interval]
-------------+----------------------------------------------------------------
       _cons |   .8164138   .0224157    36.42   0.000     .7724799    .8603478
-------------+----------------------------------------------------------------
v001         |
   var(_cons)|   .0414592   .0163845                      .0191086    .0899524
------------------------------------------------------------------------------
LR test vs. logistic model: chibar2(01) = 8.27        Prob >= chibar2 = 0.0020

. estat icc

Intraclass correlation

------------------------------------------------------------------------------
                       Level |        ICC   Std. Err.     [95% Conf. Interval]
-----------------------------+------------------------------------------------
                        v001 |   .0124453   .0048571      .0057748    .0266146
------------------------------------------------------------------------------
```

7. The conclusion section doesn’t make any concrete recommendations please specify it. Please add a recommendations or policy implications section.

**Response: The conclusion has been rewritten to reflect the reviewer’s comments to incorporate some recommendations and policy implications (see pages 24—25)**

**Reviewer #2**

8. However, without contesting the veracity of this result, there is clearly a bias in the impact of the women’s age on the probability of presenting a positive risk. What would be the incidence of the mother’s age on her probability risk among mothers aged 33 or less?

**Response: The definition of “high-risk fertility behavior” is used in many papers**^[2]^ **and in real life, sometimes it is not easy to avoid “social bias”. For instance, we know that the risk of dying is closely related to age, as it is in this case, but all studies on human mortality cannot bypass “age” which is key to understand the distribution of the outcome across ages.**

9. The manuscript is technically sound, however, as to the geographical distribution of risky behavior, it is difficult to know whether the authors are talking about the Northern region or the Eastern region.

**Response: Thank you for this comment. If the reviewer looks closely to the Democratic Republic of the Congo (DRC) map, it is clear that North-East part of the country has been devastated, and likely the displacement of populations could explain the distribution of high spots of high-risk fertility behaviours. We kindly ask the reviewer to look in Figures 4 and 6. Clearly, the East region (including the provinces of North Kivu, South Kivu and Maniema) is more affected.**

10. The authors argue that:” the country has been devastated by successive wars and armed conflict since 1996 in its Eastern part. As a consequence, the health system has worsened, especially in the Eastern region of the country. This has certainly increased the prevalence of high-risk fertility behaviors in the country”. But their findings reveal that higher risk are mostly prevalent in the northern part of the country. Why are the high spots of fertility risk not in the Eastern region? Which factors do explain the high spots in the Northern part of the country

**Response: This is a follow up question regarding the geographical distribution of high-risk fertility behavior of women of reproductive ages in the Democratic Republic of the Congo (DRC). Again, Figures 4 and 6 are very informative. In the DRC, the East region includes the provinces of North Kivu, South Kivu and Maniema. There is no confusion at all, these Figures clearly show that these provinces are more affected. To some extent, the Northern region is also affected and this is likely due to the displacement of populations in the country.**

11. The literature that studies the relationship between political instability, wars and fertility decisions seems to disagree on the nature of the relationship between armed conflict and fertility choices. On one hand, we have a group of studies that believes that political instability and related violence, not to mention economic uncertainties, negatively affect social returns such as population growth, and so it will logically follow a decline in fertility decisions and therefore less predominant risky fertility behaviors ((Berrebi and Ostwald (2015), Caldwell (2004); Agadjanian et al (2011), McGinn (2000)). On the other hand, we have authors who rather think that armed conflicts would be likely to reinforce fertility decisions (Urdal and Che (2013), McGinn et al (2011)…), and thus increase risky fertility behaviors .
    This article is not precise enough at this level, since it is difficult to identify if it is in the first or the second group. Indeed, the text is a bit ambiguous. It seems to indicate a strong prevalence of risky fertility behaviors in the North of the country.
    This article would therefore stand out from the existing literature by identifying what is happening in the case of the Republic Democratic of Congo in the Eastern region of the country which has been affected by the war. And even more by identifying the mechanisms Through which the violence experienced by this region would reinforce the risky fertility behaviors. Highlighting such mechanisms could indeed be interesting targets for policy makers.

**Response: The authors thank the reviewer for time and devotion to comment on the paper. However, there are differences between “fertility intentions” to which the reviewer is referring to and “observed fertility” on which the definition of “high-risk fertility behavior (HRFB)” is framed. The manuscript is not “positional” paper with regards to previous studies as the reviewer mentioned. Rather, the paper empirically tested the geographical distribution of HRFB and associated factors. Cross-sectional studies like this one can detect, in short-run, negative social returns such as population growth which is a long-run effect. This paper provides a tentative explanation of the distribution of HRFB given findings from spatial analyses.**

**Reviewer #3**

12. The definition of high-risk fertility behaviour is not discussed in detail. For instance, the authors decide that having 4 at least four children puts the woman in a “high-risk fertility behaviour category”. Having a birth above age 34 is also considered “high-risk”. I think the authors should provide literature to support these choices. Considering these behaviours as high risk is also very much context-dependent, and this should be discussed.

**Response: Thank you for this. Discussions to this question are provided in detail in comment #8 from Reviewer #2. In the paper, literature cited to support this definition, and some papers are already published in PLOS ONE (see comment #8)**

13. The measurement is also debatable. As far as I understand (but I found it was not clear in the manuscript), young women (e.g. less than 35) are by definition less likely to be in the “high-risk” category, since they will not (yet) have had children above age 34. In the same vein, young women are less likely to be in the “high-risk category”, since they are less likely to have had 4 children. Overall, I found this indicator not to be specific (many dimensions are mixed), and very much related to the age of the women.

**Response: Thank you for this comment on which we could continue to reflect. This is related to comment #12 (Reviewer #3) and comment #8 (Reviewer #2). The measurement of the outcome variable “high-risk fertility behavior (HRFB)” is not mixed at all since the definition is clear. To be in HRFB category, three criteria should be met: (*i*) age at last birth (below 18 years OR above 34 years); (*ii*) birth interval (less than 24 months); and (*iii*) birth order (above 3 children). A woman is defined as “HRFB” if she meets at least one of the three criteria. In some papers, scholars have distinguished between single HRFB and multiple HRFB.**^[3]^ **The definition used in the paper is the most used in the literature and as such is highly justifiable.**

14. The identification of risk factors is not convincing. There are interesting results, but it looks like a series of variables was used in the regression model without theoretical justification. One finds some surprising results (e.g. for the standard of living), and the authors do not convincingly explain these results. In my opinion, this may be due to the fact that many variables are included in the model, some being endogenous. I do not understand why the variable one “decision to use contraception” is used in the model. The variable “ANC visits” is included. Does this mean that only ever-pregnant women are in the sample?

**Response: Thank you for this comment and we will provide a sound justification of the selection of independent variables included in the analyses. In the sub-section “Measures”, under “independent variables”, the manuscript list six references as theoretical and empirical background of the paper. I understand that the reviewer should have welcomed a “theory” but authors are also limited by journal’s requirements in terms of the length of the paper. They are therefore obliged to squeeze the paper to comply with journal’s requirements.**

15. I could not figure out if the authors restricted their analyses to women in union. I suppose so since the partner’s level of education is used, but it is not clearly mentioned, and the consequence of this selection on the results is not discussed. N should be mentioned in Table 1.

**Response: It is clearly said in the “Abstract” that the study is about 11,497 “married women” of reproductive ages in the Democratic Republic of the Congo. The analytical strategy adopted to include only “complete cases”. The original definition of high-risk fertility behavior**^[4]^ **included only “married women” and this paper adopted this definition to keep only married/cohabiting women. This also answers the question regarding the selection of women on the overall results. The sample size (N) is now included in Table 1.**

16. Are community-level variables measured for primary sampling units?

**Response: Most studies using Demographic and Health Surveys (DHS) data define the specific “community” level depending upon the information available. In the paper, this is the “cluster” level as collected in DHS data. Using the information available, community-level variables were defined/built for this paper. For instance, the variable “community literacy level” was built using the information about literacy for each woman in the cluster. At individual level, valid responses included 0 “cannot read at all”; 1 “able to read only parts of the sentence”, and 2 “able to read the whole sentence”. First, the variable is recoded “1” if the woman is “able to read the whole sentence”, and “0”, otherwise. Second, the “average” is computed for each cluster (v001), and three quantiles are to define three “community literacy” categories: “1” Low; “2” Medium; and “3” High level. The same thinking was done with community socioeconomic status built using the household wealth index in the cluster.**

17. Some surprising results deserve more discussion (partner’s level of education, household wealth index). Can this be due to collinearity (even if the VIF is not very high, this can be a substantive issue)?

**Response: Following this reviewer’s comments, additional analyses (adding/removing variables) were conducted but didn’t change the magnitude of the findings. In the manuscript, we have provided a tentative justification of findings. (See page 24—25)**

18. I think the authors could improve the justification for testing interactions. I think they are not convincingly justified. Figures 1 and 2 could be dropped or moved to an appendix. Figure 3 is a nice figure but clearly shows that age is a major factor in high-risk fertility.

**Response: Most papers about high-risk fertility behaviors never tested for the interactions addressed in the current manuscript. Figures 1—3 are major contribution of the paper in the existing literature about high-risk fertility behavior (HRFB) in developing countries. This interaction can be further tested in other countries for robustness checks. Figure 1 tests the interaction between women’s education and place of residence. The authors are convinced that place of residence is of chief importance to fully captured the effect of education of HRFB. In Figure 2, the manuscript adds another layer of place of residence with respect to age. Clearly, the odds of HRFB is higher among women in rural areas at a given age. This finding has never been clearly mentioned in previous literature. The authors think they convey sound information.**

19. This is not surprising given that having a birth above 35 is in the definition of HRFB. Figure 5 could be dropped. It looks like the output of a software package which is not discussed.

**Response: Figure 5 informs the readers about “spatial autocorrelation” and it is clearly mentioned in the manuscript. Most studies with “spatial analyses” component generally informs the readers about the relevance of “autocorrelation” whether there is a “clustering effect” of the outcome.**

20. Spatial analyses offer interesting insights, but the description of the methods does not allow for understanding them in detail. In addition, I think the authors should select one or two approaches (e.g. the hot spots, or the interpolation). Figure 6 and Figure 7 are very interesting, but we expect some more discussion for these hotspots.

**Response: Thank you for this comment. The authors have added more details about Figures 6 and 7 (See pages 23—24). Regarding the choice of the methods, just to mention that each method responds to a specific analytical need. Therefore, they are all justified in this study.**

21. In addition, what are the implications, from a programmatic point of view?

**Response: Following reviewer #1 (comment #7), authors have added programmatic implications of the findings in the paper (see pages 24—25)**

**References**

1.	Tessema ZT, Tamirat KS. Determinants of high-risk fertility behavior among reproductive-age women in Ethiopia using the recent Ethiopian Demographic Health Survey: a multilevel analysis. *Trop Med Health*. 2020; 48:93. <https://doi.org/10.1186/s41182-020-00280-1>.

2.	Tamirat KS, Tesema GA, Tessema ZT. Determinants of maternal high-risk fertility behaviors and its correlation with child stunting and anemia in the East Africa region: A pooled analysis of nine East African countries. *PLOS ONE*. 2021; 16:e0253736. <https://doi.org/10.1371/journal.pone.0253736>.

3.	Tessema ZT, Azanaw MM, Bukayaw YA, Gelaye KA. Geographical variation in determinants of high-risk fertility behavior among reproductive age women in Ethiopia using the 2016 demographic and health survey: a geographically weighted regression analysis. *Arch Public Health*. 2020; 78:74. <https://doi.org/10.1186/s13690-020-00456-5>.

1. For more details, see Price et al. (2005). “Power Analysis in Survey Research: Importance and Use for Health Educators”. American Journal of Health Education, 36(4), pp. 202—207
2. See for more details ^1–3^, and <https://dhsprogram.com/data/Guide-to-DHS-Statistics/High-Risk_Fertility_Behavior.htm>
3. See for instance ^2^. In this paper however, HRFB was treated as a key independent variable.
4. <https://dhsprogram.com/data/Guide-to-DHS-Statistics/High-Risk_Fertility_Behavior.htm>
