# Supplementary material for: High-risk fertility behaviours among women of reproductive ages in the Democratic Republic of the Congo: Prevalence, correlates, and spatial distribution
Source: PLoS One. 2023 Mar 16;18(3):e0283236. doi: 10.1371/journal.pone.0283236 (PMC10019681; doi:10.1371/journal.pone.0283236)
Supplement: Attachment — Submitted filename: Point-by-point Responses Feb 2023.docx [file pone.0283236.s003.docx]

**Point-by-point responses—Revision 2**

**Additional Editor Comments:**

Although all reviewers agreed that this version of your manuscript has greatly improved, review 2 raised a crucial issue. You did not adequately address their comments. Critically address all comments and acknowledge the limitations of the study.
In addition to a point-by-point response, track or highlight all changes to your revised manuscript.

**Response: Thank you for this, but I think you meant Reviewer #3 after a careful reading of the comments below. I would say that as an Academic Editor and reviewers of Plos One, and many other journals, I take Reviewers’ comments very seriously. Reviewers are doing an incredible job to improve the quality of publications, and overall, the quality of Science. That said and with respect, I can’t escape reviewers’ comments. In our last point-by-point response, we addressed all comments from reviewer #3 (this revision). We will respond again to his comments, hoping that will clear all questions and suggestions he has made. And we appreciate his help to improve the paper.**

**Review #3 (previous and current comments)**

Reviewer #3: Dear authors. Thank you for your replies. You clarified some of the issues that were raised, but I find several were not addressed, or in a very brief way.

1. The definition of high-risk fertility behaviour is not discussed in detail. For instance, the authors decide that having 4 at least four children puts the woman in a “high-risk fertility behaviour category”. Having a birth above age 34 is also considered “high-risk”. I think the authors should provide literature to support these choices. Considering these behaviours as high risk is also very much context-dependent, and this should be discussed.

**Response: The definition of “high-risk fertility behavior” is used in many papers**^[1]^ **and in real life, sometimes it is not easy to avoid “social bias”. For instance, we know that the risk of dying is closely related to age, as it is in this case, but all studies on human mortality cannot bypass “age” which is key to understand the distribution of the outcome across ages.**

2. The measurement is also debatable. As far as I understand (but I found it was not clear in the manuscript), young women (e.g. less than 35) are by definition less likely to be in the “high-risk” category, since they will not (yet) have had children above age 34. In the same vein, young women are less likely to be in the “high-risk category”, since they are less likely to have had 4 children. Overall, I found this indicator not to be specific (many dimensions are mixed), and very much related to the age of the women.

**Response: Thank you for this comment on which we could continue to reflect. The measurement of the outcome variable “high-risk fertility behavior (HRFB)” is not mixed at all since the definition is clear. To be in HRFB category, three criteria should be met: (*i*) age at last birth (below 18 years OR above 34 years); (*ii*) birth interval (less than 24 months); and (*iii*) birth order (above 3 children). A woman is defined as “HRFB” if she meets at least one of the three criteria. In some papers, scholars have distinguished between single HRFB and multiple HRFB.**^[2]^ **The definition used in the paper is the most used in the literature and as such is highly justifiable.**

3. The identification of risk factors is not convincing. There are interesting results, but it looks like a series of variables was used in the regression model without theoretical justification. One finds some surprising results (e.g. for the standard of living), and the authors do not convincingly explain these results. In my opinion, this may be due to the fact that many variables are included in the model, some being endogenous. I do not understand why the variable one “decision to use contraception” is used in the model. The variable “ANC visits” is included. Does this mean that only ever-pregnant women are in the sample?

**Response: Thank you for this comment and we will provide a sound justification of the selection of independent variables included in the analyses. In the sub-section “Measures”, under “independent variables”, the manuscript list six references as theoretical and empirical background of the paper. I understand that the reviewer should have welcomed a “theory” but authors are also limited by journal’s requirements in terms of the length of the paper. They are therefore obliged to squeeze the paper to comply with journal’s requirements.**

4. I could not figure out if the authors restricted their analyses to women in union. I suppose so since the partner’s level of education is used, but it is not clearly mentioned, and the consequence of this selection on the results is not discussed. N should be mentioned in Table 1.

**Response: It is clearly said in the “Abstract” that the study is about 11,497 “married women” of reproductive ages in the Democratic Republic of the Congo. The analytical strategy adopted to include only “complete cases”. The original definition of high-risk fertility behavior**^[3]^ **included only “married women” and this paper adopted this definition to keep only married/cohabiting women. This also answers the question regarding the selection of women on the overall results. The sample size (N) is now included in Table 1.**

5. Are community-level variables measured for primary sampling units?

**Response: Most studies using Demographic and Health Surveys (DHS) data define the specific “community” level depending upon the information available. In the paper, this is the “cluster” level as collected in DHS data. Using the information available, community-level variables were defined/built for this paper. For instance, the variable “community literacy level” was built using the information about literacy for each woman in the cluster. At individual level, valid responses included 0 “cannot read at all”; 1 “able to read only parts of the sentence”, and 2 “able to read the whole sentence”. First, the variable is recoded “1” if the woman is “able to read the whole sentence”, and “0”, otherwise. Second, the “average” is computed for each cluster (v001), and three quantiles are to define three “community literacy” categories: “1” Low; “2” Medium; and “3” High level. The same thinking was done with community socioeconomic status built using the household wealth index in the cluster.**

6. Some surprising results deserve more discussion (partner’s level of education, household wealth index). Can this be due to collinearity (even if the VIF is not very high, this can be a substantive issue)?

**Response: Following this reviewer’s comments, additional analyses (adding/removing variables) were conducted but didn’t change the magnitude of the findings. In the manuscript, we have provided a tentative justification of findings. (See page 24—25)**

**The invaluable contribution of this paper is its focus on one country. Although principles can be universal, context-specific effects/associations could be observed. Even within the same country, urban-rural differences or differences across provinces/regions could be observed. That’s why scholars need to be carefully of overgeneralization. That’s also why I personally don’t encourage pooling data to increase statistical power and overlooking country and regional differences. In my opinion, policy implications drawn from this kind of studies have less programmatic implications because conclusions drawn from a specific country are not necessarily transferable.**

7. I think the authors could improve the justification for testing interactions. I think they are not convincingly justified. Figures 1 and 2 could be dropped or moved to an appendix. Figure 3 is a nice figure but clearly shows that age is a major factor in high-risk fertility.

**Response: Most papers about high-risk fertility behaviors never tested for the interactions addressed in the current manuscript. Figures 1—3 are major contribution of the paper in the existing literature about high-risk fertility behavior (HRFB) in developing countries. This interaction can be further tested in other countries for robustness checks. Figure 1 tests the interaction between women’s education and place of residence. The authors are convinced that place of residence is of chief importance to fully captured the effect of education of HRFB. In Figure 2, the manuscript adds another layer of place of residence with respect to age. Clearly, the odds of HRFB are higher among women in rural areas at a given age. This finding has never been clearly mentioned in previous literature. The authors think they convey sound information.**

8. This is not surprising given that having a birth above 35 is in the definition of HRFB. Figure 5 could be dropped. It looks like the output of a software package which is not discussed.

**Response: The starting point of spatial is to “better justify” the necessity of undertaking spatial analyses with cross-sectional data. The first statistic of interest is “Moran’s *I*” which is an inferential tool, indicating whether there is clustering or dispersion of the outcome of interest, in this case “HRFB”. In this paper, Figure 5 informs the readers about “spatial autocorrelation” and this is clearly mentioned in the manuscript.**

**Figure 5 has been reworked to eliminate the software appearance and details have been added accordingly, as previously mentioned (see pages 17—18)**

9. Spatial analyses offer interesting insights, but the description of the methods does not allow for understanding them in detail. In addition, I think the authors should select one or two approaches (e.g. the hot spots, or the interpolation). Figure 6 and Figure 7 are very interesting, but we expect some more discussion for these hotspots.

**Response: Thank you for this comment. The authors have added more details about Figures 6 and 7 (See pages 23—24). Regarding the choice of the methods, just to mention that each method addresses a specific analytical need. Therefore, they are all justified in the current study.**

**More specifically, spatially analyses follow a certain logic that we respected in this paper. In fact, the paper is interested in equal distribution of HRFB in the country. Therefore, the starting point was to see if there is even distribution of HRFB. The response was “NO”. Therefore, the next question was “is there sign of spatial clustering?” Fig 5 answers this question and clearly, there is sign of clustering given the statistics reported (Page 17: z-score of 3.62; p-value = 0.000297). Finally, the hotspot analysis aimed at identifying these clusters (Figs. 6—7).**

10. In addition, what are the implications, from a programmatic point of view?

**Response: Following reviewer #1 (comment #7), authors have added programmatic implications of the findings in the paper (see pages 24—25)**

11. I think the restriction of the sample to "married women with at least one live birth" could be better justified. Why don't you use women who have never had a live birth? With your approach, most women will be in the high-risk category, unless they are between 19 and 34.

**Response: To make things simple, the paper extends existing literature within the specific context of the Democratic Republic of the Congo. The paper, along the point-by-point responses, have extensively explained the three criteria to be met to be included in at-risk category: (*i*) age at last birth (below 18 years OR above 34 years); (*ii*) birth interval (less than 24 months); and (*iii*) birth order (above 3 children). The HRFB was defined in the context of marriage, and extended to cohabiting women. This paper adopted this definition/categorisation to define HRFB.**

**The reviewer commented on the inclusion of “women who have never had a live birth”. The essence of the outcome itself excludes women who have never had a live birth because they cannot meet the three criteria mentioned above.**

12. Why is age included in this way in the models (Table 2)? By definition, women less than 19 and over 34 will be in the high-risk category? The effect of age on the log odds is thus by definition not linear. Could you include age as a categorical variable?

**Response: Just to put things in context, and going back to the definition of HRFB. The “age” included in the definition of HRFB is the age at last birth while “age at survey” is included as independent variable.**

**Now, to respond to the reviewer, age was included as continuous variable. There is no justifiable rationale to include it as categorical variable, and this approach (or decision) has been criticized in the statistical literature.^[4]^ This reference provides an interesting discussion on how to treat continuous variable in logistic regression. Clearly, scholars think it is not a good idea to categorize a continuous variable. The same conclusion can be found in Ranganathan et al. (2017).**^[5]^ **In this paper, we quoted this, on page 151: “For continuous data (e.g., age, height, or weight), it is tempting to divide the subjects into categories (e.g., age >50 years vs. age ≤50 years). This is not a good practice since the cutoffs tend to be arbitrary and part of the information is lost.”**

**Finally, the reviewer should recall that the manuscript points out to same conclusion, when he said “the effect of age on the log odds is thus by definition not linear is thus by definition not linear”. This is one of the contribution of the paper in testing interactions between women’s age at survey and place of residence (see Fig 2).**

How can you explain the "positive" effect of wealth on high-risk fertility behaviour, especially in model 1? You mention "This finding might reflect the associations observed between husband/partner’s education and high-risk fertility behaviours". I do not understand what you mean.

**Response: In the Discussion section (p. 23), we mentioned that (1) these findings were unexpected; however, that’s what the data returned. This reminds us that, as we mentioned before, each country is unique and pooling data can be misleading. (2) At some point, having many children comes with cost that better-off households and highly educated husbands/partners are more able to handle. In fact, the idea is that highly educated husbands/partners are more likely to live in better-off households, given these findings.**

Most previous comments still hold (Figure 5 not useful, not much justification for the variables, etc.).

**Response: In this point-by-point response, we have (re)taken all questions from last review. Hope this clears all grey area which remained from our last answers.**

Reviewer #4: The opening statement in the abstract about SDG is not necessary as there is no discussion about it afterwards.

**Response: Thank you for the comment. The statement has been dropped in the abstract.**

The statement about "culture and social development" in the abstract is not necessary since they do not represent any unit of analysis in this study.

**Response: Thank you for the comment. The fact is that sub-Saharan Africa countries are not homogeneous culturally and economically. That might explain some differences observed between countries.**

Line 112 says "theoretically": it is hard to know what it means in this context as there is no reference to any theory. Maybe the authors mean studies. Check.

**Response: To avoid any confusion, the word “theoretically” was deleted. Thank you for this suggestion.**

The study limitations should be expanded to include "non-interrogation of the possible contribution of armed conflict" and "age of the respondents (women's).

**Response: Thank you for the comment. This has been incorporated in the limitations section (page 24—25).**

Aside this, the authors have adequately addressed the reviewers' comments.

**Response: Thank you**

1. See for more details ^1–3^, and <https://dhsprogram.com/data/Guide-to-DHS-Statistics/High-Risk_Fertility_Behavior.htm>
2. See for instance ^2^. In this paper however, HRFB was treated as a key independent variable.
3. <https://dhsprogram.com/data/Guide-to-DHS-Statistics/High-Risk_Fertility_Behavior.htm>
4. <https://stats.stackexchange.com/questions/60149/should-i-categorise-my-continuous-variable-for-use-in-binary-logistic-regression>
5. Ranganathan P, Pramesh CS, Aggarwal R. Common pitfalls in statistical analysis: Logistic regression. Perspect Clin Res 2017;8:148-51.
